# Repetitive arm functional tasks after stroke (RAFTAS): a pilot randomised controlled trial

**DOI:** 10.1186/s40814-016-0088-5

**Published:** 2016-08-17

**Authors:** Lianne Brkic, Lisa Shaw, Frederike van Wijck, Richard Francis, Christopher Price, Anne Forster, Peter Langhorne, Caroline Watkins, Helen Rodgers

**Affiliations:** 1 Stroke Research Group, Institute of Neuroscience, Newcastle University, 3-4 Claremont Terrace, Newcastle upon Tyne, NE2 4AE UK; 2 Institute for Applied Health Research and School of Health and Life Sciences, Glasgow Caledonian University, Cowcaddens Road, Glasgow, G4 0BA UK; 3 Academic Unit of Elderly Care and Rehabilitation, Bradford Royal Infirmary, University of Leeds and Bradford Institute for Health Research, Duckworth Lane, Bradford, BD9 6RJ UK; 4 Academic Section of Geriatric Medicine, Royal Infirmary, Floor 2, New Lister Building, Glasgow, G31 2ER UK; 5 Clinical Practice Research Unit, School of Nursing and Caring Sciences, University of Central Lancashire, Brook 419, Preston, PR1 2HE UK

**Keywords:** Feasibility study, Randomised controlled trial, Stroke, Upper limb rehabilitation, Repetitive functional task practice

## Abstract

**Background:**

Repetitive functional task practise (RFTP) is a promising treatment to improve upper limb recovery following stroke. We report the findings of a study to determine the feasibility of a multi-centre randomised controlled trial to evaluate this intervention.

**Methods:**

A pilot randomised controlled trial recruited patients with new reduced upper limb function within 14 days of acute stroke from three stroke units. Participants were randomised to receive a four week upper limb RFTP therapy programme consisting of goal setting, independent activity practise, and twice weekly therapy reviews in addition to usual post stroke rehabilitation, or usual post stroke rehabilitation. The recruitment rate; adherence to the RFTP therapy programme; usual post stroke rehabilitation received; attrition rate; data quality; success of outcome assessor blinding; adverse events; and the views of study participants and therapists about the intervention were recorded.

**Results:**

Fifty five eligible patients were identified, 4-6 % of patients screened at each site. Twenty four patients participated in the pilot study. Two study sites met the recruitment target of 1–2 participants per month. The median number of face to face therapy sessions received was 6 [IQR 3–8]. The median number of daily repetitions of activities recorded was 80 [IQR 39–80]. Data about usual post stroke rehabilitation were available for 18/24 (75 %). Outcome data were available for 22/24 (92 %) at one month and 20/24 (83 %) at three months. Outcome assessors were unblinded to participant group allocation for 11/22 (50 %) at one month and 6/20 (30 %) at three months. Four adverse events were considered serious as they resulted in hospitalisation. None were related to study treatment. Feedback from patients and therapists about the RFTP programme was mainly positive.

**Conclusions:**

A multi-centre randomised controlled trial to evaluate an upper limb RFTP therapy programme provided early after stroke is feasible and acceptable to patients and therapists, but there are issues which need to be addressed when designing a Phase III study. A Phase III study will need to monitor and report not only recruitment and attrition but also adherence to the intervention, usual post stroke rehabilitation received, and outcome assessor blinding.

**Trial registration:**

International Standard Randomised Controlled Trials Number (ISRCTN) 58527251

## Background

Loss of arm function affects 69 % of people who have a stroke [1]. Only 12 % of stroke patients with initial upper limb motor impairment regain full function [2]. Stroke patients who are unable to use their arm may require long term support from their families or social services. Patients report that rehabilitation pays insufficient attention to arm recovery and they have identified optimising arm function as a research priority [3]. It is currently unclear how to maximise arm recovery after stroke. A systematic review of upper limb therapy interventions suggests that patients benefit most from exercise programmes in which functional tasks are directly practised, rather than interventions which are impairment focused, such as muscle strengthening [4].

Functional or task specific practice is underpinned by the movement science approach to stroke rehabilitation [5]. Repetitive functional task practice (RFTP) seeks to enhance motor learning by undertaking practice of functionally relevant tasks [6, 7]. Other key components of RFTP include: intensity of practice; active cognitive involvement; and feedback on performance [5]. A Cochrane overview of systematic reviews found moderate quality evidence that arm function following a stroke can be improved by repetitive task training [8]. However, included studies were small, often did not describe the interventions in detail, and several had methodological weaknesses [9–20]. The authors highlighted the need for further high quality randomised controlled trials to strengthen this evidence [8].

We aimed to establish the feasibility of a Phase III multi-centre randomised controlled trial designed to determine the clinical effectiveness of an upper limb RFTP therapy programme for acute stroke patients. The objectives of the pilot study were to report: the recruitment rate; adherence to the upper limb RFTP therapy programme; the usual post stroke rehabilitation received by study participants; the attrition rate; data quality; the success of outcome assessor blinding; adverse events; and the views of study participants and therapists about the intervention.

## Methods

### Study design

**Fig. 1:**
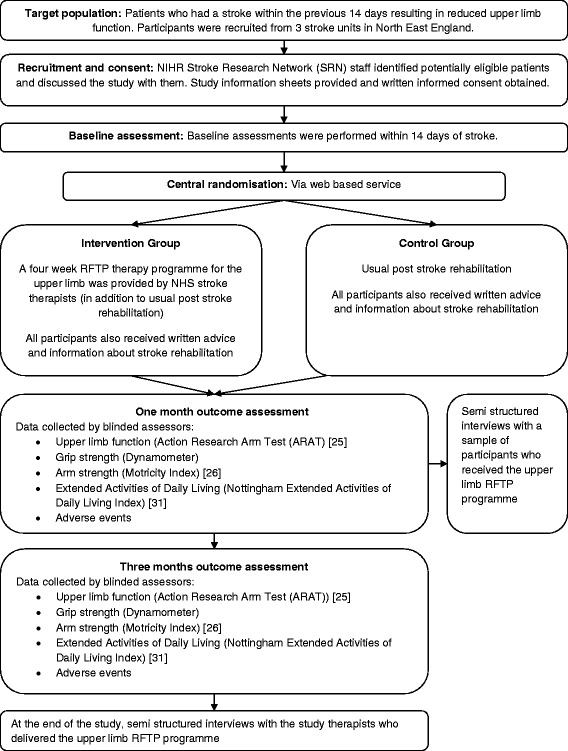
Flowchart of the RAFTAS study design

We conducted a multi-centre pilot randomised controlled trial to inform the design of a Phase III study to evaluate an upper limb RFTP therapy programme for acute stroke patients. The Medical Research Council (MRC) guidance was followed to develop and evaluate the intervention [21]. A flowchart of the study design is shown in Fig. 1.

### Study setting

Three National Health Service (NHS) stroke units in North East England participated in the pilot study. All provided in-patient acute stroke care and rehabilitation on a stroke unit and had community therapy follow up services. They were typical of stroke services which would be invited to participate in a multi-centre study.

### Participants

We aimed to recruit patients with a recent first-ever or recurrent stroke resulting in new reduced upper limb function. Inclusion criteria were: age ≥ 18 years; within 14 days of stroke onset; new reduced upper limb function but with retained ability to lift the affected hand any distance off their lap; capable of undertaking the upper limb RFTP therapy programme and adhering to the study protocol; able to provide informed consent to participate in the study; and home address within the community services catchment area of a participating study site. Exclusion criteria were: unable to follow the upper limb RFTP programme e.g. due to cognitive impairment or receptive aphasia; other significant upper limb impairment e.g. fixed contracture, frozen shoulder, severe arthritis, and upper limb pain that inhibited participation in the upper limb RFTP therapy programme; and a diagnosis likely to interfere with rehabilitation e.g. registered blind, receiving palliative care. As this was a pragmatic study, we did not use standardised scales to define any inclusion or exclusion criteria. Inclusion and exclusion criteria were based upon clinical opinion reflecting how patients would be identified to receive the upper limb RFTP programme in clinical practice. Screening was undertaken by NHS research support staff at each site who were Good Clinical Practice (GCP) certified and trained by in study methods and procedures by the study physiotherapist (LB).

### Recruitment and consent

Potentially eligible patients were approached by NHS research support staff who discussed the study with them and provided a study information sheet. After allowing sufficient time for this information to be considered, written consent was obtained from patients who wished to take part.

### Baseline assessment

The following data were collected prior to randomisation by NHS research support staff: demographic data; time from stroke; first-ever or recurrent stroke; stroke type (infarct or haemorrhage); stroke subtype (total anterior circulation stroke (TACS), partial anterior circulation stroke (PACS), lacunar stroke (LACS), posterior circulation stroke (POCS)) [22]; stroke severity (National Institutes of Health Stroke Scale (NIHSS)) [23]; pre-stroke handicap (Oxford Handicap Scale (OHS)) [24]; hand dominance; arm function (Action Research Arm Test (ARAT)) [25], arm strength (Motricity Index) [26], and grip strength (dynamometer).

### Randomisation

Participants were randomised within 14 days of acute stroke to receive the upper limb RFTP therapy programme (in addition to usual post stroke rehabilitation) or usual post stroke rehabilitation in a 1:1 allocation ratio. A central independent web based randomisation service hosted by Newcastle University Clinical Trials Unit was used. Participants were stratified according to study centre to ensure that intervention and control group participants were evenly distributed across study centres.

### Development of the upper limb RFTP therapy programme

We reviewed the theoretical basis of RFTP and the structure and content of the upper limb RFTP therapy programmes which had been evaluated in previous research [9–17]. The upper limb RFTP therapy programme was then developed in collaboration with stroke physiotherapists and occupational therapists. Feedback was sought from clinicians, stroke patients and carers. The therapy programme was then refined before being tested in a clinical setting. Prior to undertaking the pilot randomised controlled trial, the study physiotherapist who developed the programme (LB) delivered the upper limb RFTP therapy programme to seven stroke patients in two of the participating stroke units. As this was provided in addition to usual post stroke rehabilitation, she worked closely with participants’ local NHS therapists, ward teams and community teams to ensure that care was coordinated. Feedback from study participants and the experience gained by the study physiotherapist were used to further refine the upper limb RFTP therapy programme. The study physiotherapist developed and provided a face to face training programme with regular updates and a manual to enable local NHS therapists to deliver the programme in the pilot study.

### Intervention: the upper limb RFTP therapy programme

The upper limb RFTP therapy programme was a four week programme of twice daily self-practised RFTP for patients with new reduced upper limb function early after stroke led by local NHS therapists (a physiotherapist or occupational therapist). The programme comprised of functional tasks, embedded in routine everyday activities completed on the ward or at home. This aimed to make the programme highly relevant to participants, promote ‘carry over’ into real life situations, and encourage self-practise.

With the support of local NHS therapists, participants selected and practised functional tasks, termed recovery activities, which involved goal-focused upper limb movement sequences. Recovery activities related to categories identified from the most popular participant-selected goals in previous studies: washing, dressing and eating/drinking [27, 28]. There was also an optional category which enabled participants to select an activity which was not listed under the other categories (e.g. using a mobile phone). Optional tasks offered choice and aimed to enhance participant motivation.

Participants were asked to practise each selected recovery activity independently for up to 20 times, twice per day, for four weeks. For participants who were unable to attempt a full task e.g. picking up a cup, recovery activities were divided into part tasks e.g. reaching towards the cup and practised in the same way as full tasks. The duration and intensity of the upper limb RFTP therapy programme were based upon the interventions used in previous RCTs of RFTP [9–17], the feasibility of delivering the intervention in the NHS, and feedback from stroke patients and carers in the development phase.

At the start of the programme, the local NHS therapist performed a routine clinical assessment to determine the patient’s upper limb impairment and to identify other neurological deficits that may impact on upper limb function (e.g. inattention or cognitive deficit). The participant identified their two most important upper limb rehabilitation priorities and these were used to set two functional rehabilitation goals which were potentially achievable within the four week programme. The therapist and participant then selected two recovery activities from lists created for each category. A wide range of recovery activities was available in each category and they were ordered into three levels of ability which were generated by considering sensorimotor demands (e.g. the amount of upper limb movement and coordination required) and the level of mental processing needed to complete the activity. The local NHS therapist demonstrated the chosen recovery activities and ensured that the participant was confident to practise them independently.

Intervention group participants were given an individualised upper limb RFTP participant handbook. The handbook included guidance about undertaking their chosen recovery activities, along with sections to log their twice daily practice and to provide feedback about the upper limb RFTP therapy programme. The participant handbook also included a section with advice and information concerning stroke recovery and care of their affected upper limb. The local NHS therapist demonstrated how to use the handbook and how to complete the activity log sheets and feedback sections.

Participants were reviewed by the local NHS therapist twice per week in hospital or at home. These sessions consisted of a clinical re-assessment of the participant’s affected upper limb impairment and review of progress towards their chosen functional goals. The goals and/or recovery activities were adjusted according to progress. If the participant had achieved a goal, a new goal was set and a new recovery activity was selected. If the participant found a goal or recovery activity too challenging, alternatives were chosen. If a participant regained normal upper limb function based upon the clinical assessment of the therapist and achieved all of their upper limb rehabilitation goals before the end of the four week intervention period, they were discharged from the programme. Otherwise, participants received a final therapy review at the end of the four week programme either in hospital or at home. The final review included a discussion about the participant’s future goals and advice about maintaining upper limb function.

### Control treatment

In the Phase III trial, as we wished to assess the effectiveness of the intervention in addition to current upper limb RFTP programme clinical practice, usual post stroke rehabilitation was chosen as the control treatment. Participants randomised to receive control treatment also received a study handbook, prepared by the study team, which contained advice and information about stroke, rehabilitation and positioning of the arm and hand after stroke. The duration and content of routine physiotherapy and occupational therapy provided to intervention and control group participants for 4 weeks post randomisation were recorded by local NHS therapists on a structured proforma. We acknowledge that there is currently no standard approach to upper limb rehabilitation post-stroke as rehabilitation is tailored to the needs of each individual and is dependent upon the availability of local resources [29]. The National Clinical Guideline for Stroke recommends a minimum 45 min of each appropriate therapy for 5 days per week [30].

### Outcome assessments

Outcomes were assessed at one month (+/− 3 days) and 3 months (+/− 5 days) following randomisation. These time points will be used in a Phase III study to look at the treatment effect at the end of the intervention period and to report the longer term effectiveness of the upper limb RFTP therapy programme. The following data were collected: arm function (Action Research Arm Test (ARAT)) [25]; grip strength (dynamometer); arm strength (Motricity Index) [26]; extended activities of daily living (Nottingham Extended Activities of Daily Living Index) [31].

### Blinding

Outcome assessors were four therapists from stroke units or community services. They were not involved in the direct care of the participants and were intended to be blinded to treatment allocation. As the upper limb RFTP therapy programme involved regular practise and assessment, it was not possible to blind stroke patients or local NHS therapists who treated the patient to treatment allocation. After each assessment, the assessor was asked to record whether they had become unblinded. We attempted to blind stroke unit staff to treatment allocation by means of the study handbooks which were given to both control and intervention groups and were identical in external appearance.

### Adverse events

The safety of the upper limb RFTP programme was evaluated by examining the occurrence of adverse events in accordance with National Research Ethics Committee (NRES) guidance for non Clinical Trial of an Investigational Medicinal Product (CTIMP) trials [32]. To collect adverse event data, participants were asked at each outcome assessment if they had any new medical problems. Participants were also asked specifically about upper limb pain and fatigue using visual analogue scales, and muscle tone in the upper limb was assessed by the Modified Ashworth Scale [33].

### Feedback from intervention group participants and therapists

Intervention group participants were asked to provide feedback about the upper limb RFTP therapy programme on their activity log sheets. They were also asked open questions about their experiences and opinions about the therapy programme at therapy reviews by local NHS therapists. The study physiotherapist (LB) undertook semi-structured 1:1 interviews with a convenience sample of patients when they left/completed the therapy programme and with the three main local NHS therapists who had delivered the upper limb RFTP programme. These data were coded and categorised into positive and negative comments and themes.

### Sample size and statistical analysis plan

As this was a pilot study a formal sample size calculation was not performed.

We aimed to recruit 60 participants, based on 1–2 participants per month, from three study centres recruiting for 1 year. This is the level of recruitment expected per site for multi-centre stroke rehabilitation trials in the UK [34]. Analysis of pilot studies should be mainly descriptive [35, 36]. Numbers and percentages were used for categorical variables. Mean and standard deviation (SD) or median and interquartile range (IQR) were reported for continuous variables. As data are available from larger studies of upper limb interventions post stroke, which recruited participants from a similar patient population and used the same validated outcome measures, we did not seek to use data from the pilot study to inform the sample size calculation for a larger trial.

## Results

**Fig. 2:**
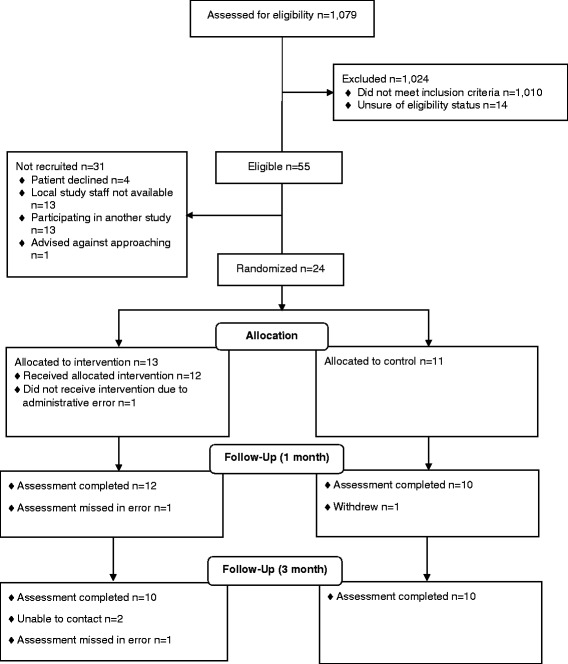
RAFTAS CONSORT flow diagram

The consolidated standards of reporting trials (CONSORT) flow diagram is shown in Fig. 2.

### Screening and recruitment

The planned recruitment period was 1 year but preparing study documents and seeking approvals took longer than anticipated. The actual recruitment period was 03.06.13–28.02.14. Site A was open to recruitment for 30 weeks, site B 37 weeks, and site C 38 weeks. One thousand and seventy nine patients were screened, and 55 eligible patients were identified. This was between 4 and 6 % of patients screened at each site.

The main recorded reason why patients were not eligible was that they had no new reduced upper limb function: 206/1010 (20 %). One hundred and eighty one patients (18 %) were thought to be unable to comply with the upper limb RFTP programme because of speech or cognitive problems, and 147 (15 %) lived outside the catchment area for community follow up visits. The reason for exclusion was not recorded for 337/1010 (33 %).

Twenty four of the 55 (44 %) eligible patients took part in the study. Site A recruited four participants, site B nine participants and site C 11 participants. There was a wide variation in the proportion of eligible patients recruited between sites. Site C recruited 11/11 (100 %) eligible patients, site B recruited 9/23 (39 %) and site A 4/21 (19 %). Potentially eligible patients did not participate because 13 were already participating in another study which did not allow co-enrolment (site A); a local NHS therapist was not available to provide the RFTP programme to seven patients (four site A and three site B); NHS research support staff were not available to consent six patients (site B); and a consultant advised against approaching one patient (site B). Four patients (site B) declined to take part in the study: one felt that the upper limb RFTP therapy programme would be too difficult; one felt that there was insufficient content; and two did not give a reason. Two of the three study sites were able to recruit the target of 1–2 participants per month.

### Characteristics of the study population

Table 1 shows the baseline characteristics of study participants. Participants were randomised a median of 5 (IQR 2–11) days after stroke and had reduced upper limb function with a median ARAT of 20 (IQR 3–35). As could be anticipated in a small pilot study, intervention and control groups were not well-matched at baseline. Intervention group participants were older, had less severe strokes with milder upper limb impairment and function and were randomised earlier than control group participants.

**Table 1:** Baseline characteristics of participants

	Intervention group	Control group
	*n* = 13	*n* = 11
Gender		
Male *n* (%) Female *n* (%)	8 (61.5 %) 5 (38.5 %)	9 (81.8 %) 2 (18.2 %)
Age		
Median (IQR) years Missing	71 [67–78] 2	65 [57–72] 1
Pre-stroke handicap (Oxford Handicap Scale [24])		
0 1 2 3 Range 0–5: no symptoms – severe handicap	12 (92.3 %) 0 1 (7.7 %) 0	5 (45.5 %) 3 (27.3 %) 2 (18.2 %) 1 (9.1 %)
First ever stroke Missing	8 (61.5 %) 0	8 (72.7 %) 0
Dominant hand affected by stroke Missing	5 (38.5 %) 0	7 (63.6 %) 0
Time from stroke to randomisation		
Median (IQR) days Missing	6 [2.5–11.5] 0	4 [2–9] 0
Stroke type		
Assumed infarct (no clinically relevant infarct on CT) Clinically relevant infarct on CT/MRI Intracerebral haemorrhage Missing	0 (0 %) 11 (85 %) 0 (0 %) 2 (15 %)	3 (27 %) 5 (46 %) 1 (9 %) 2 (18 %)
Stroke sub-type (*n* %) [22]		
Total anterior circulation syndrome (TACS) Partial anterior circulation syndrome (PACS) Lacunar stroke (LACS) Posterior circulation stroke (POCS) Missing	1 (8 %) 2 (15 %) 8 (62 %) 0 (0 %) 2 (15 %)	1 (9 %) 5 (46 %) 4 (36 %) 1 (9 %) 0 (0 %)
Stroke severity (National Institutes of Health Stroke Scale [23])		
Median [IQR]) Missing Range 0–42: no symptoms – severe impairment	3 [2–5] 1	6 [3–7] 0
Arm function (Action Research Arm Test [25])		
Median (IQR) Missing Range 0–57: no movement – full function	32 [10–37] 0	8 [1–22] 0
Arm strength (Motricity Index) [26]		
Median (IQR) Missing Range 0–100: no movement – normal strength	73 [48–77] 2	40 [29–52] 0
Grip strength (dynamometer)		
Median (IQR) kg Missing	12 [4–21] 2	7 [2–18] 0

### Intervention

Seven local NHS therapists (four physiotherapists and three occupational therapists) were trained to deliver the upper limb RFTP therapy programme. Of 13 intervention group participants, four received the intended eight face-to-face therapy sessions. Two were discharged early from the programme as per protocol as they had achieved all of their upper limb therapy goals and had regained full upper limb function. One participant did not wish to undertake recovery activities and was discharged from the therapy programme. Two participants (site A) were reported to have received the upper limb RFTP therapy programme but documents were not returned to the coordinating centre so it is unclear how many face to face sessions they received. A further three participants did not receive all eight sessions and the reasons for this are unclear (site A *n* = 1, site C *n* = 2). One patient did not receive any of the upper limb RFTP therapy programme as the local NHS therapist was not informed that the patient was participating in the study (site B). The median number of therapy sessions delivered per patient was 6 (IQR 3–8).

Sixty five upper limb rehabilitation goals were selected. The goals related to: dressing *n* = 18 (28 %); washing *n* = 17 (26 %); eating/drinking *n* = 17 (26 %); 13 (20 %) were in the optional category and related to activities such as writing, handling money, kitchen activities and playing cards. Sixty (92 %) goals were achieved during the study. Participants returned activity log sheets for a median of 20 (IQR 2.5–24) days. Participants were asked to record the number of repetitions for each recovery activity. The intended maximum number of repetitions per day was 80 (20 repetitions of two activities, twice per day). The median number undertaken daily was 80 (IQR 39–80, range 0–150) for the 11 participants whose activity log sheets were returned.

### Usual post stroke rehabilitation

Information about usual post stroke rehabilitation was available for 17/24 (71 %) participants. Data were available for a median of 8 (IQR 6–19.5) days per participant for intervention group participants and 5 (IQR 3.5–11.5) days for control participants. Unfortunately we did not record when participants were discharged from usual post stroke rehabilitation. The content of therapy sessions was recorded for 238 sessions for intervention group participants and 94 sessions for control group participants. The intervention group/control participant sessions comprised: mobility 100 (42 %) vs 43 (46 %); upper limb RFTP 21 (9 %) vs 8 (9 %); other upper limb rehabilitation 53 (22 %) vs 21 (22 %); activities of daily living 37 (16 %) vs 13 (14 %); and other 27 (11 %) vs 9 (10 %).

### Attrition

Follow up at 1 month was 22/24 (92 %): one participant withdrew from the study and one outcome assessment was missed. Follow up at 3 months was 20/24 (83 %). Three participants could not be contacted and one was overlooked in error.

### Outcome measures

Outcome measures at 1 and 3 months are shown in Table 2.

**Table 2:** Clinical outcomes at 1 and 3 months

	Intervention 1 month	Control 1 month	Intervention 3 months	Control 3 months
	*n* = 13	*n* = 11	*n* = 13	*n* = 11
Arm function (ARAT) [25]				
Median (IQR) Missing	55 [38–57] 1	46 [29–57] 2	57 [50–57] 3	48 [35–57] 1
Grip strength (dynamometer)				
Median (IQR) kg Missing	15 [8–20] 3	11 [5–26] 1	13 [5–21] 4	14 [4–28] 1
Arm strength (Motricity Index) [26]				
Median (IQR) Missing	91 [76–99] 1	79 [55–91] 1	88 [65–99] 3	88 [72–94] 1
Nottingham Extended Activities of Daily Living Scale [31]				
Median (IQR) Missing	36 [10–54] 5	34 [25–46] 2	43 [9–60] 3	52 [32–58] 2

The median ARAT score at one month was 55 (IQR 38–57) for the intervention group and 46 (IQR 29–57) for the control group. At 3 months, the median ARAT score for the intervention group was 57 (IQR 50–57) and control group 48 (35–57). The maximum score achievable on the ARAT is 57. Levels of missing data were acceptable.

### Blinding

Outcome assessors reported that they were unblinded to participant group allocation for 11/22 (50 %) at 1 month 6/20 (30 %) at 3 months. The same assessor carried out one and three month assessments for 20/21 (95 %) participants, so some assessors had forgotten that they felt unblinded at one month when they undertook the 3-month assessment. Because of the large number of staff providing care at each stroke unit, we did not try to measure the success of group concealment among stroke unit staff.

### Adverse events

Four adverse events were considered serious as they resulted in hospitalisation: two falls, one episode of postural hypotension and one episode of gastritis. All were considered unrelated to the upper limb RFTP therapy programme. Seventeen adverse events were reported, 10 in the intervention group and seven in the control group. All adverse events were considered unrelated to the intervention.

Participants were specifically asked about upper limb pain, fatigue and assessed for increased muscle tone in their affected arm. At 1 month 5/13 (38 %) participants in the intervention group and 6/11 (55 %) participants in the control group reported pain in the upper limb affected by stroke. At 3 months, these corresponding data were 5/13 (38 %) in the intervention group and 5/11 (45 %) in the control group. Nearly all participants reported some degree of fatigue at 1 and 3 months. At 1 month 5/13 (38 %) participants in the intervention group and 5/11 (45 %) participants in the control group had increased upper limb tone. At 3 months these corresponding data were 3/13 (23 %) in the intervention group and 5/11 (45 %) in the control group.

### Feedback from intervention group participants and therapists

During the 4-week upper limb RFTP therapy programme, participants recorded 107 positive comments about the programme on their therapy log sheets and 39 negative comments. Positive comments reported that the programme was enjoyable, challenging and motivating. Negative comments predominantly related to fatigue, although some participants found the programme too challenging. At twice weekly therapy reviews, nine participants gave positive comments to their local NHS therapist about the upper limb RFTP therapy programme, reporting that they felt they were benefiting from the programme. Eight participants gave negative comments about the programme which again predominantly related to fatigue. One participant did not enjoy participating in the upper limb RFTP therapy programme and another felt that it aggravated a back problem. All of the seven participants who provided feedback at the end of the upper limb RFTP therapy programme to their NHS therapist felt that it was reasonable to start early after stroke. Six felt that being reviewed twice per week by the local NHS therapist was about right, and one felt that this was not enough. Six found the participant handbook helpful. All found goal setting useful. Three participants took part in a 1:1 semi-structured interview with the study physiotherapist upon completing the upper limb RFTP therapy programme. No points were raised that had not been identified previously.

The three therapists who had the most experience of delivering the upper limb RFTP therapy programme participated in semi-structured interviews at the end of the study. They provided positive feedback about the upper limb RFTP programme and gave some suggestions about minor changes to study documents.

## Discussion

We have demonstrated that a multi-centre randomised controlled trial to determine the clinical effectiveness of the upper limb RFTP therapy programme is feasible, but there are a number of issues which need to be addressed in the design and delivery of a Phase III study.

NHS site selection will be an important issue. One of the strengths of the pilot study was that it was a multi-centre study undertaken in sites which are typical of sites which are likely to participate in a Phase III study. Pilot studies are often undertaken in a single centre where the chief investigator is based, with strong local ownership and engagement of clinical and research teams. This can lead to over-optimism about the feasibility of a multi-centre study.

We have obtained valuable insight about issues which are likely to be encountered in a Phase III study and gained understanding about the type and amount of support which sites are likely to need from the study coordinating centre. Multi-centre stroke rehabilitation trials are relatively rare, and two of the three sites had limited experience of stroke rehabilitation research. In selecting sites and throughout a Phase III study, we will need to ensure that key individuals within the stroke unit and community stroke teams are committed to the trial and are able to deliver the study as per protocol. It may be helpful to have several linked sites within a region which are supported by a local study coordinator.

The recruitment target of 1–2 participants per month was met by two of the three study sites. It would not have been possible for sites to have more than two intervention participants at any one time because of the additional work load for local NHS therapists. We felt that it was important that the upper limb RFTP therapy programme was delivered by the local NHS therapists rather than research therapists so that the intervention was evaluated as it would be delivered in clinical practice. Funding for the upper limb RFTP programme was a NHS excess treatment cost [37]. The excess treatment cost was the cost of the upper limb RFTP programme over and above usual post stroke rehabilitation. Excess treatment costs are not research costs funded by a grant, but are costs funded by the normal NHS commissioning process for stroke rehabilitation services. Our experience of leading multi-centre stroke rehabilitation trials over the last 10 years is that there is wide variation between NHS organisations in their approach to NHS excess treatment costs. Some NHS organisations provide additional funding to individual therapists or rehabilitation services to deliver study treatments, whilst others agree for study treatments to be undertaken within the current service budget. In this pilot study, the programme was delivered by local NHS therapists in addition to their usual work load and therapists did not always have dedicated time to provide the intervention and to complete study documents. In selecting sites for a Phase III study, we will need to consider their approach to excess treatment costs and the views of local therapists about delivering the upper limb RFTP programme within the local excess treatment costs policy. The approach of a NHS organisation to excess treatment costs may impact upon whether or not a site agrees to participate in the study, the delivery of the intervention, and data quality about the intervention and usual post stroke care.

Although the recruitment target was met in two of the three sites, a large proportion of eligible patients were not enrolled (56 %) and there was a wide variation between sites. A number of eligible patients were not approached at site A as they were participating in studies which did not allow co-enrolment. These were hyperacute and acute drug studies. Provided that there are no potential interactions between interventions, and assessments are not too burdensome, patients should be offered the opportunity to participate in a second study. In selecting sites for a Phase III study, we will need to determine the compatibility of our study with the site’s portfolio of research studies and discuss co-enrolment with the chief investigators of ongoing studies.

Another reason for non-enrolment of potentially eligible patients relates to the lack of availability of NHS research support staff to recruit participants and local NHS therapists to deliver the upper limb RFTP therapy programme. Two sites had no prospective cover for absence. Although there was more than one NHS therapist trained to deliver the upper limb RFTP therapy programme at each site, when a member of the therapy team was away, the time available for research activities was reduced, so patients could not be randomised. There was no ring fenced resource to provide study treatments, reflecting the reality of undertaking multi-centre rehabilitation studies within the NHS. The Phase III study design will need to allow for the impact holidays, sick leave, change of staff etc. upon recruitment and delivery of the intervention.

Our eligibility criteria were pragmatic and based upon clinical judgement, as would be used to decide whether or not to provide the treatment in clinical practice. We may need to include arm function measured by the ARAT as an eligibility criterion for a Phase III study [25]. This measure of arm function is likely to be our primary outcome as it is well-validated and is widely used in studies evaluating upper limb rehabilitation post stroke. The maximum ARAT score is 57 and the minimum clinically important difference is 6 points [38]. Because of a ceiling effect, we will need to consider excluding patients who score 52 or above at baseline so that we will be able to detect this change at 1 and 3 months (three participants in the pilot study scored 52 or more at the baseline assessment). It would have been helpful to have assessed the inter-rater reliability of the ARAT and other key scales within the pilot study. This work will need to be undertaken during a Phase III study.

Participants were willing to take part in the study within 14 days of acute stroke and felt that this was a reasonable time to be approached. Intervention and control groups were not well matched at baseline. This is likely to be due to a small sample size and should not be an issue in a larger study. As it is important that groups are balanced at baseline in terms of severity of upper limb function, participants will be stratified by this parameter at randomisation in a Phase III study.

The intervention has been carefully developed with patient and carer involvement at all stages. Participants were able to practise recovery activities themselves as per protocol with twice weekly review by a therapist both in hospital and at home.

The study manual and supporting documents can be used in a Phase III study and adhere to TIDieR (template for intervention description and replication) [39].

There is wide variation in clinical practice regarding the amount and content of upper limb rehabilitation provided, so in a future study, there is a need to accurately record the amount and content of the usual post stroke rehabilitation received by participants in both randomisation groups. As there were a number of non-returned or poorly completed therapy forms in the pilot study, methods of minimising this need to be included in a Phase III study. More information was available about the usual post stroke rehabilitation received by intervention group participants than those in the control group, which could be a source of bias about the amount and content of therapy received. The forms used to record the amount and content of the intervention and usual post stroke rehabilitation will be reviewed by the study team and NHS therapists to see if they can be simplified and/or reduced. We need to develop a robust system of ensuring their return to the study coordinating centre, with regular checks for data completeness and data quality. Alternatively, an electronic system could be developed to collect data with reminders and prompts but additional resource would likely be needed to support local data entry. We also need to stress in our training programme for local NHS therapists the importance of obtaining high-quality data.

The timing and content of outcome assessments will remain unchanged in a Phase III study. The attrition rate observed in the pilot study and completeness of key outcome measures are acceptable for a stroke rehabilitation study but could be improved. In a Phase III trial, we will consider using electronic prompts and reminder letters for outcome assessors to try to prevent assessments being missed. We will review methods to try to prevent participants being lost to follow-up by seeking information about discharge destination when a participant leaves hospital.

Lack of blinding can result in numerous sources of bias. Particular risks for stroke rehabilitation trials are resentful demoralisation of participants randomised to the control group [40] and competitive therapy bias, where therapy staff may feel that patients in the control group are disadvantaged and subsequently provide them with increased rehabilitation [41]. We did consider developing an attention control treatment, but this would have added to the complexity and cost of the trial. We were disappointed that a large number of assessments was unblinded at the 1- and 3-month outcome assessments. Unfortunately, we did not collect data about when and how outcome assessors became unblinded and in retrospect this would have been useful. All outcome assessors worked within the stroke unit and community stroke services at the site where they undertook assessments. For a Phase III study, we will consider employing outcome assessors who work out with the stroke service.

The upper limb RFTP therapy programme was acceptable to patients early after stroke and local NHS therapists and the majority of goals were achieved. A number of patients in both intervention and control groups experienced fatigue. This is likely to be stroke related rather than specific to the intervention as there is a high prevalence of fatigue following stroke [42]. Fatigue needs to be taken into account when considering rehabilitation goals. During a Phase III study, an Independent Data Monitoring and Ethics Committee (IDMEC) will monitor differences between intervention and control groups in levels of fatigue. No concerns about the safety of the upper limb RFTP programme were identified in the pilot study, and the safety reporting system can be used in a multi-centre study.

## Conclusions

A multi-centre randomised controlled trial to evaluate an upper limb RFTP therapy programme provided early after stroke is feasible and acceptable to patients and therapists, but there are issues which need to be addressed when designing a Phase III study. A Phase III study will need to monitor and report not only recruitment and attrition, but also adherence to the intervention, usual post stroke rehabilitation received and outcome assessor blinding. Because of issues found in the pilot study, a Phase III multi-centre study will require an internal pilot study with stop/go rules based upon recruitment rate, adherence to the intervention, attrition and completeness of outcome assessments. The internal pilot study will determine if the actions taken to address the issues raised in the current pilot study have been successful.

## Abbreviations

ARAT, action research arm test; CONSORT, consolidated standards of reporting trials; CTIMP, clinical trial of an investigational medicinal product; GCP, good clinical practice; IDMEC, independent data monitoring and ethics committee; IQR, interquartile range; ISRCTN, International standard randomised controlled trials number; LACS, Lacunar stroke; MRC, medical research council; NHS, National Health Service; NIHSS, National Institutes of Health Stroke Scale; NRES, National Research Ethics Committee; OHS, Oxford Handicap Scale; PACS, partial anterior circulation stroke; POCS, posterior circulation stroke; RAFTAS, repetitive arm functional tasks after stroke; RCT, randomised controlled trial; RFTP, repetitive functional task practise; SD, standard deviation; SRN, stroke research network; TACS, total anterior circulation stroke; TIDieR, template for intervention description and replication; VISTA, the virtual international stroke trials archive
